# Comparison of Cartesian and Non-Cartesian Real-Time MRI Sequences at 1.5T to Assess Velar Motion and Velopharyngeal Closure during Speech

**DOI:** 10.1371/journal.pone.0153322

**Published:** 2016-04-13

**Authors:** Andreia C. Freitas, Marzena Wylezinska, Malcolm J. Birch, Steffen E. Petersen, Marc E. Miquel

**Affiliations:** 1 NIHR Cardiovascular Biomedical Research Unit at Barts, William Harvey Research Institute, Queen Mary University of London, London, United Kingdom; 2 Clinical Physics, Barts Health NHS Trust, London, United Kingdom; Birkbeck College, UNITED KINGDOM

## Abstract

Dynamic imaging of the vocal tract using real-time MRI has been an active and growing area of research, having demonstrated great potential to become routinely performed in the clinical evaluation of speech and swallowing disorders. Although many technical advances have been made in regards to acquisition and reconstruction methodologies, there is still no consensus in best practice protocols. This study aims to compare Cartesian and non-Cartesian real-time MRI sequences, regarding image quality and temporal resolution trade-off, for dynamic speech imaging. Five subjects were imaged at 1.5T, while performing normal phonation, in order to assess velar motion and velopharyngeal closure. Data was acquired using both Cartesian and non-Cartesian (spiral and radial) real-time sequences at five different spatial-temporal resolution sets, between 10 fps (1.7×1.7×10 mm^3^) and 25 fps (1.5×1.5×10 mm^3^). Only standard scanning resources provided by the MRI scanner manufacturer were used to ensure easy applicability to clinical evaluation and reproducibility. Data sets were evaluated by comparing measurements of the velar structure, dynamic contrast-to-noise ratio and image quality visual scoring. Results showed that for all proposed sequences, FLASH spiral acquisitions provided higher contrast-to-noise ratio, up to a 170.34% increase at 20 fps, than equivalent bSSFP Cartesian acquisitions for the same spatial-temporal resolution. At higher frame rates (22 and 25 fps), spiral protocols were optimal and provided higher CNR and visual scoring than equivalent radial protocols. Comparison of dynamic imaging at 10 and 22 fps for radial and spiral acquisitions revealed no significant difference in CNR performance, thus indicating that temporal resolution can be doubled without compromising spatial resolution (1.9×1.9 mm^2^) or CNR. In summary, this study suggests that the use of FLASH spiral protocols should be preferred over bSSFP Cartesian for the dynamic imaging of velopharyngeal closure, as it allows for an improvement in CNR and overall image quality without compromising spatial-temporal resolution.

## Introduction

Velopharyngeal insufficiency (VPI) is a speech impairment resulting from the incomplete closure between the soft palate (velum) and the posterior and lateral pharyngeal walls, i.e. the velopharyngeal port. As a result, air escapes through the nasal cavity during phonation and patients most commonly present hypernasal speech [1]. Clinical assessment of VPI primarily depends on the speech therapists’ perceptual evaluation. Imaging is usually performed using x-ray videofluoroscopy and/or nasendoscopy [2]. X-ray videofluoroscopy provides satisfactory visualization of the hard palate and pharyngeal walls, however, soft tissue contrast (e.g. velum) is relatively poor. To improve contrast, a suspension of barium is usually applied to the vocal tract mucosae [3,4]. However, this renders the procedure more unpleasant which is a major constraint in younger patients [4]. Additionally, repetitive exposure to ionizing radiation is of concern. Nasendoscopy consists of the passage of a fiber-optic scope trans-nasally into the nasopharynx, providing an en-face view of the velopharyngeal port. The introduction of the scope is rather invasive and requires full patient cooperation. Younger patients or those with a deviated nasal septum usually require local anesthetic to the nostril [1]. Additionally, wide-angle distortions and enlarged adenoids may hamper closure assessment [2].

The limitations of both techniques have strongly supported the use of dynamic MRI in speech research, as summarized in a review of the field [5]. MRI provides tomographic images with improved soft tissue contrast, ideal for vocal tract imaging, in multiple image planes without repositioning the patient. An increased number of studies have used real-time MRI to image the upper airway during speaking, singing and swallowing, both in healthy [6–15] and VPI individuals [4,16–19]. Comparison of the performance of real-time MRI with videofluoroscopy revealed good correspondence when assessing the velopharyngeal closure pattern [4].

Despite the advantages, clinical implementation still faces many challenges. Velopharyngeal closure is characterized by the rapid transition of the velum between rest and elevated position; reported between 50 and 150 ms per cycle [20] depending on speech sample and rate. To reliably capture velar motion, previous studies [9,10] have suggested a temporal resolution of 20 frames per second (fps). Although experiments with VPI patients seem to suggest that all closure events during normal phonation are detected at around 10 fps, lower frame rates lead to increased blurring and missed closure events [21]. Additionally, Sagar et al. [16] reported overestimation of the velopharyngeal gap size and misleading diagnostic evaluation when comparing MRI to videofluoroscopy, due to the low frame rate used (2 fps). In summary, temporal resolution is a key issue when assessing velopharyngeal closure and careful consideration should be given to the acquisition frame rate. However, temporal acceleration is limited by the trade-off in signal-to-noise ratio (SNR), spatial resolution and overall image quality.

Most research on clinical scanners has been focussed on Cartesian acquisitions. Turbo spin echo (TSE) “zoom” techniques with partial Fourier acquisition have been used to achieve between 4–6 fps with spatial resolutions of 1.5×3.1×6 mm^3^ [4] and 3.9×1.9×6 mm^3^ [12]. Rapid gradient-echo sequences, both spoiled (like the fast low angle shot (FLASH) sequence) and steady state free precession (SSFP) Cartesian sequences, have also been frequently used in dynamic vocal tract imaging [6–8,13,16,22]. These have been most commonly implemented with parallel imaging techniques, both image based (SENSE [23]) and k-space based (GRAPPA [24]), to further improve temporal resolution. Vocal tract configuration during singing and swallowing was imaged at 10 fps (1.7×2.7×6 mm^3^) with a FLASH acquisition and GRAPPA [7,8,22]. Scott et al. [6] investigated velopharyngeal closure at various spatial-temporal resolutions (10 fps, 1.9×1.9×10 mm^3^ to 20 fps, 2.7×2.7×10 mm^3^) at both 1.5T and 3T using SSFP sequences and SENSE. Also, Martins et al. investigated velar movement in European Portuguese vowels with a Cartesian FLASH sequence and GRAPPA reconstruction, imaging the vocal tract at 14 fps (3.3×1.6×8 mm^3^) [13]. These studies have successfully covered a wide range of speech tasks and clinical applications with standard scanning resources, however image quality and temporal resolution is still limited. Additionally, non-Cartesian acquisitions and nonstandard reconstruction methods have been suggested to improve spatial-temporal resolution [9–11,25–29].

Narayanan et al. [10,30] imaged the vocal tract using an interleaved spiral acquisition, this allowed an acquired frame rate of about 9 fps (2.7×2.7×5 mm^3^), reconstructed up to 24 fps with a sliding window method [31]. Since each frame is acquired in multiple segments, frame rate can be improved by reconstructing each image with the most recent set of spirals’ interleaves. However, no additional information is added to the raw data and temporal fidelity is reliant on native frame rate. Improved native temporal resolution (21 fps, 1.9×1.9×6.5 mm^3^) was later reported by Bae et al. [9] using a multi-shot FLASH spiral protocol with regional saturation, where saturation bands are applied to eliminate the signal outside the chosen field-of-view (FOV). Niebergall et al. [11] performed imaging of the vocal tract at 30 fps (1.5×1.5×10 mm^3^) using a radial FLASH acquisition with a nonlinear inversion reconstruction method [32]. Although this allowed for improved image quality at higher undersampling factors than SENSE or conventional gridding reconstruction, reconstruction was intrinsically more complex and time consuming. Lingala et al. suggested an optimized system for the dynamic imaging of the vocal tract using a custom-built upper airway coil, multi-shot spiral sampling and a sparse SENSE constrained reconstruction scheme [28]. Temporal resolutions of 83.3 fps (12 ms) for a single-slice and 27.7 (36 ms) for a three-slice acquisition were achieved, with improved temporal fidelity when compared to a fully sampled gridding reconstruction. Higher frame rates were recently achieved by Fu et al., demonstrating a nominal rate of 100 fps (2.2×2.2 mm^2^) based on a Partial Separability model [29]. Although these methodologies have allowed great improvement in spatial-temporal resolution of speech imaging, they are mostly reliant on off-line reconstruction methodologies and/or non-standard resources, thus hampering immediate translation to clinical evaluation.

In summary, dynamic imaging of the vocal tract with real-time MRI is still an open field of research, and there is much variability in the preferred acquisition methods used by different research groups [33]. Therefore, there is the need for a comparison of different acquisition protocols, regarding image quality and temporal resolution trade-off, which could provide with additional insight to researchers interested in the field and assist with future translation to clinical evaluation. The aim of this study is to compare different real-time sequences, Cartesian and non-Cartesian sampling, for the dynamic imaging of speech, in particular the assessment of velopharyngeal closure. It should be underlined that no direct comparison between the k-space samplings’ (Cartesian vs. non-Cartesian) performance is intended from this study, we seek instead to provide a comparison of “best practice” protocols for clinical evaluation. To ensure that any resulting protocol could be easily reproduced and adapted to clinical evaluation, only standard hardware, acquisition and reconstruction algorithms provided by the MRI scanner manufacturer were used in this study.

## Materials and Methods

### Subjects

Five adult individuals (2 males and 3 females, range 34–50 years, median 42 years) were recruited from the staff of our institution for the main study, with a further 2 for preliminary development work. All volunteers gave informed written consent according to ethics approval of NHS research ethics committee (LREC 08/H0701/30). None of the participants had any known speech, language or hearing disabilities.

### Speech task and audio recording

Subjects were imaged in the supine position while performing a speech sample consisting of counting (1 to 10), non-sense nasal verbalization (/za-na-za/, /zu-nu-zu/, /ze-ne-ze/) and sustained phonation (/a/ as in ‘arm’ and /i/ as in ‘cheese’). Participants were provided with and asked to repeat the chosen speech sample before entering the MRI examination room. As real-time MRI sequences were used in this study, the speech sample was only produced once per dynamic acquisition, unlike triggered protocols where consecutive repetition of the speech task is necessary [5]. Audio was simultaneously recorded using a fiber-optic MR-compatible microphone (FOMRI II, Optoacoustics, Or Yehuda, Israel), strapped to the coil structure and placed adjacent and parallel to the lips of each subject. A noise-cancelling algorithm was used to reduce the background scanner noise, internal to the microphone system and similar to that described elsewhere [9]. Audio recording was started simultaneously with each MRI acquisition; however, subjects were instructed through the inter-communication system when to initiate phonation in order to allow for the noise cancellation algorithm to adjust, usually between 6 to 10 seconds. Synchronization of the recorded audio and the dynamic image data was possible using a timing trigger signal available from the scanner and recorded as a second channel of the audio signal. Examples of movies generated with synchronized audio can be seen in the supporting information files.

### MRI data acquisition

Images were acquired using a 1.5T Philips Achieva (Philips Healthcare, Best, the Netherlands) R 3.2 MRI scanner (maximum 180 mT/m/ms gradient slew rate and 33 mT/m amplitude) and a 16-channel neurovascular coil. Real-time 2D mid-sagittal images of the head and upper neck were acquired. In order to optimize image quality, the shim volume was centered around the velum.

**Fig 1:**
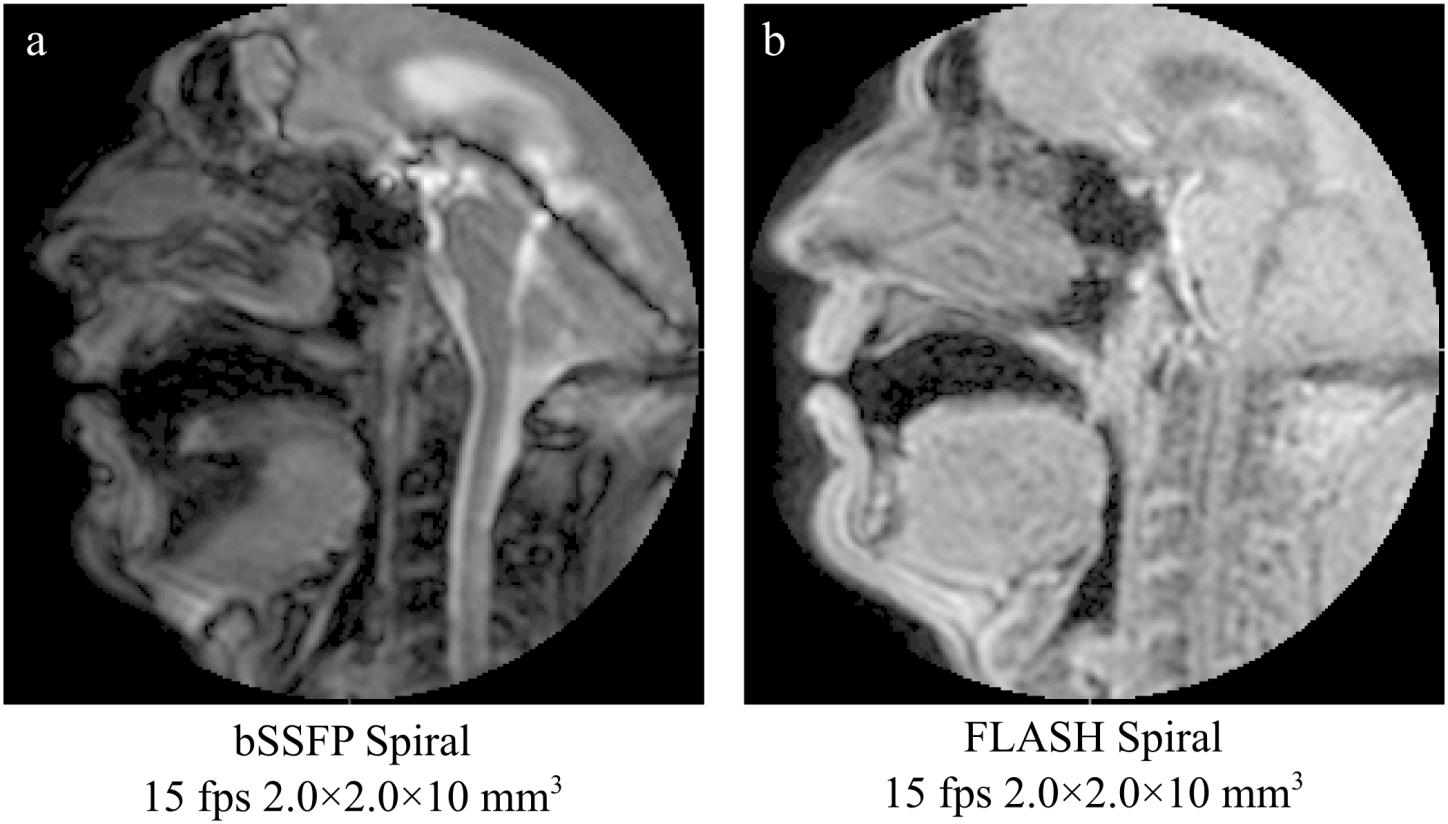
Example mid-sagittal images acquired with bSSFP Spiral and FLASH Spiral to demonstrate differences in image quality and velum blurring.

Preliminary experiments with a phantom and 2 subjects were performed to identify suitable sequences and optimize non-Cartesian acquisition. Cartesian protocols were implemented as described elsewhere [6]. Although Cartesian acquisitions were performed with balanced-SSFP sequences, preliminary testing with bSSFP non-Cartesian sequences revealed dynamic imaging with increased velum blurring and signal void artifacts due to off-resonance effects (Fig 1). Thus, FLASH-like sequences, commonly preferred in speech imaging [5], were used with the non-Cartesian acquisitions. Data acquisition for the main study was performed as follows: Cartesian protocols were implemented with a bSFFP sequence (flip angle 30°, partial Fourier factor of 0.625 and 10 mm slice thickness), while non-Cartesian protocols were implemented with a FLASH sequence (flip angle 10° and 10 mm slice thickness). Non-Cartesian sequences (sequence 1 to 3) were optimized in order to match previously implemented Cartesian protocols [6] in spatial-temporal resolution. Two additional spiral and radial sequences (sequence 4 and 5) were optimized to investigate additional spatial-temporal resolution improvement. By matching protocols in spatial and temporal resolution, a quantitative comparison in image quality performance (e.g. signal-to-noise ratio, velum distortion and presence of artifacts) between protocols could be performed. Acquisition parameters such as SENSE acceleration, “sliding window” acceleration, number of spiral interleaves, readout time and radial projections were optimized; a detailed description of used acquisition parameters is given in Table 1. Spiral protocols were implemented with 36 interleaves and a readout time of 2 ms. In order to achieve the desired temporal resolution, Cartesian acquisitions were combined with SENSE and non-Cartesian with a “sliding window” reconstruction method.

**Table 1:** Acquisition parameters at 1.5T according to sequence and acquisition sampling scheme.

Sequence	Spatial–temporal resolution	Acquisition	TE/TR (ms)	FOV (mm^2^)	Sliding window factor	SENSE factor	Radial undersampling
1	1.9×1.9 mm^2^/10fps	Cartesian	1.5/3.0	300×240	-	2.4	-
1	1.9×1.9 mm^2^ / 10 fps	Radial	2.3/5.1	180×180	5.0	-	1.57
1	1.9×1.9 mm^2^ / 10 fps	Spiral	1.0/5.1	190×190	2.0	-	-
2	2.2×2.2 mm^2^ / 15 fps	Cartesian	1.4/2.8	300×240	-	3.0	-
2	2.2×2.2 mm^2^ / 15 fps	Radial	2.1/4.7	180×180	6.0	-	1.65
2	2.2×2.2 mm^2^ / 15 fps	Spiral	1.0/5.0	190×190	3.0	-	-
3	2.7×2.7 mm^2^ / 20 fps	Cartesian	1.2/2.4	300×240	-	3.0	-
3	2.7×2.7 mm^2^ / 20 fps	Radial	1.9/4.1	180×180	6.0	-	1.62
3	2.7×2.7 mm^2^ / 20 fps	Spiral	1.0/4.8	190×190	4.0	-	-
4	1.9×1.9 mm^2^ / 22 fps	Radial	2.3/5.0	170×170	9.0	-	1.71
4	1.9×1.9 mm^2^ / 22 fps	Spiral	1.0/5.1	190×190	4.0	-	-
5	1.5×1.5 mm^2^ / 25 fps	Radial	2.7/5.9	170×170	16.0	-	1.57
5	1.5×1.5 mm^2^ / 25 fps	Spiral	1.0/6.3	190×190	6.0	-	-

Sliding window acceleration factor is defined such as per one fully acquired dynamic scan; a number of sub-dynamic scans defined by the acceleration factor are reconstructed. Therefore, frame rate is increased by the chosen sliding window factor. Radial undersampling was calculated in comparison to a radial aliasing-free sampling case (number of projections times pi/2).

### Data analysis

Dynamic images were analyzed using measurements of velum thickness and signal homogeneity, dynamic contrast-to-noise ratio (CNR) and image quality visual scoring. SPSS (v.22, IBM, New York) software was used to perform all statistical analyzes. When comparing continuous variables with multiple measurements, as in the case of CNR comparison between sequences and sampling schemes, repeated-measures one-way analysis of variance (ANOVA) was used. This was to test the null hypothesis that the mean of all samples for a certain measurement across sequences were equal, considering a significance level of 0.05. Multiple Bonferroni adjusted paired t-tests were used to identify significant pairs. Paired data sets, such as velum thickness in both velar positions, were compared using a two-tailed paired t-test. Image quality visual scoring was compared using a Kruskal-Wallis test. Significant pairs were identified with multiple pairwise comparisons of the Mann Whitney test and Bonferroni corrected significance level.

### Velum signal homogeneity and thickness

Measurements of velum thickness and signal homogeneity in the dynamic frames were carried out using OsiriX 6.0.1 32 bit (Pixmeo Sarl, Bernex, Switzerland). Velar measurements in the relaxed position were performed in frames prior to the beginning of phonation (nasal breathing) and in the elevated position, in frames corresponding to the sustained phonation of /a/. Velum thickness was measured as the distance between the velar knee and the velar dimple [34]. Signal homogeneity of the velum was measured as the ratio between the mean and the standard deviation of the signal retrieved from a region-of-interest (ROI) drawn to include the velum structure. This gives an indication of the presence of artifacts or distortion in the selected ROI, as this would result in a decrease in the calculated ratio. Since speech assessment with real-time MRI is yet to be translated into clinical practice, direct correlation between these image quality parameters and clinical relevance still needs to be fully understood. However, low signal homogeneity of the velum could indicate an image where the velum is masked or distorted by artifacts, and thus, clinical assessment of closure could be hampered.

### Intensity-time CNR

**Fig 2:**
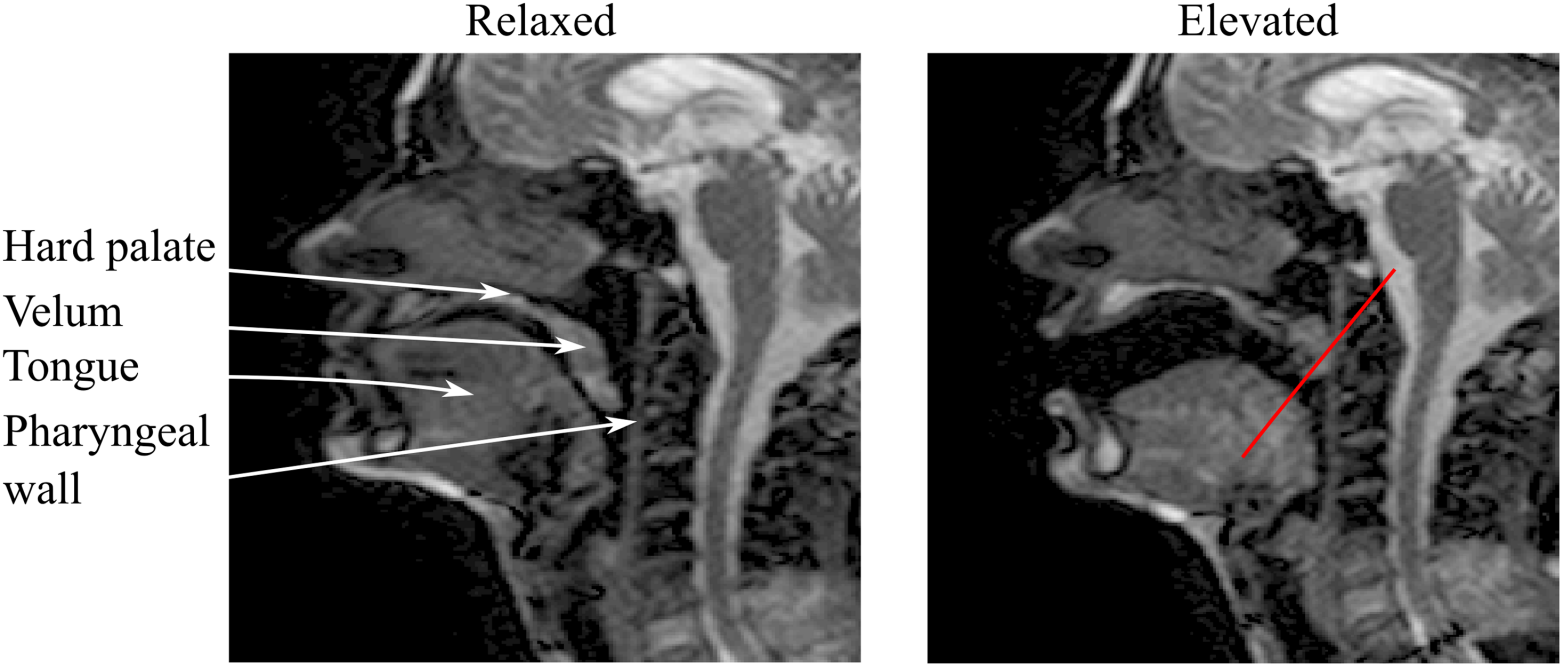
Example mid-sagittal images to demonstrate the upper vocal tract configuration at the relaxed and elevated velar positions. Image data acquired in the same subject using Cartesian sequence 1 acquisition protocol. Reference line was selected along the primary direction of motion of the velum to indicate the selected profile when generating the intensity-time plots.

CNR measurements were carried out using MATLAB (release 2014b, The MathWorks, Natick, MA). Intensity-time plots were obtained by selecting an intensity profile in each dynamic frame along a reference line (Fig 2b) and stacking profiles from adjacent time frames side-by-side. This allows for a representation of velar motion throughout acquisition, where the horizontal direction is representative of time. CNR was measured in a section of the intensity-time plots, considering two ROIs selected over the velum and adjacent oral cavity, as follows:

$$CNR=\frac{S_{velum}-S_{oral\;cavity}}{\sigma_{oral\;cavity}}$$ (1)

Where *S*_*velum*_ is the mean signal in the ROI drawn in the velum, *S*_*oral cavity*_ and *σ*_*oral cavity*_ are the mean and standard deviation signal in the ROI drawn in the neighboring oral cavity.

### Visual scoring

**Fig 3:**
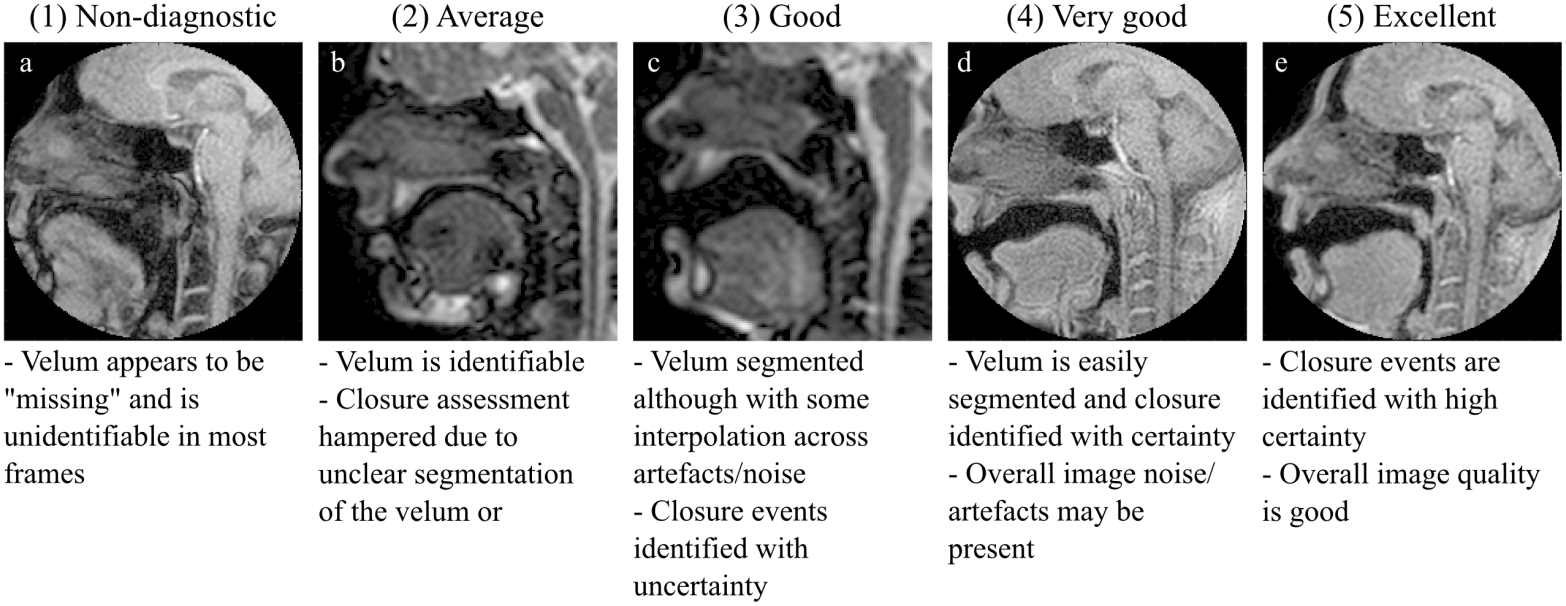
Five-point scoring scale from non-diagnostic (a) to excellent (e) image used to visually score image quality.

Image quality of the dynamic data was scored visually. Images were rated blindly and randomly using a five-point scale by two independent observers (imaging physicists) with, 2 and 20 years of MRI experience. For intra-observer reliability data, observer 1 also scored the images a second time, approximately one month after the first scoring. Further details on the chosen scoring scale can be seen in Fig 3.

## Results

### Velum signal homogeneity and thickness

**Fig 4:**
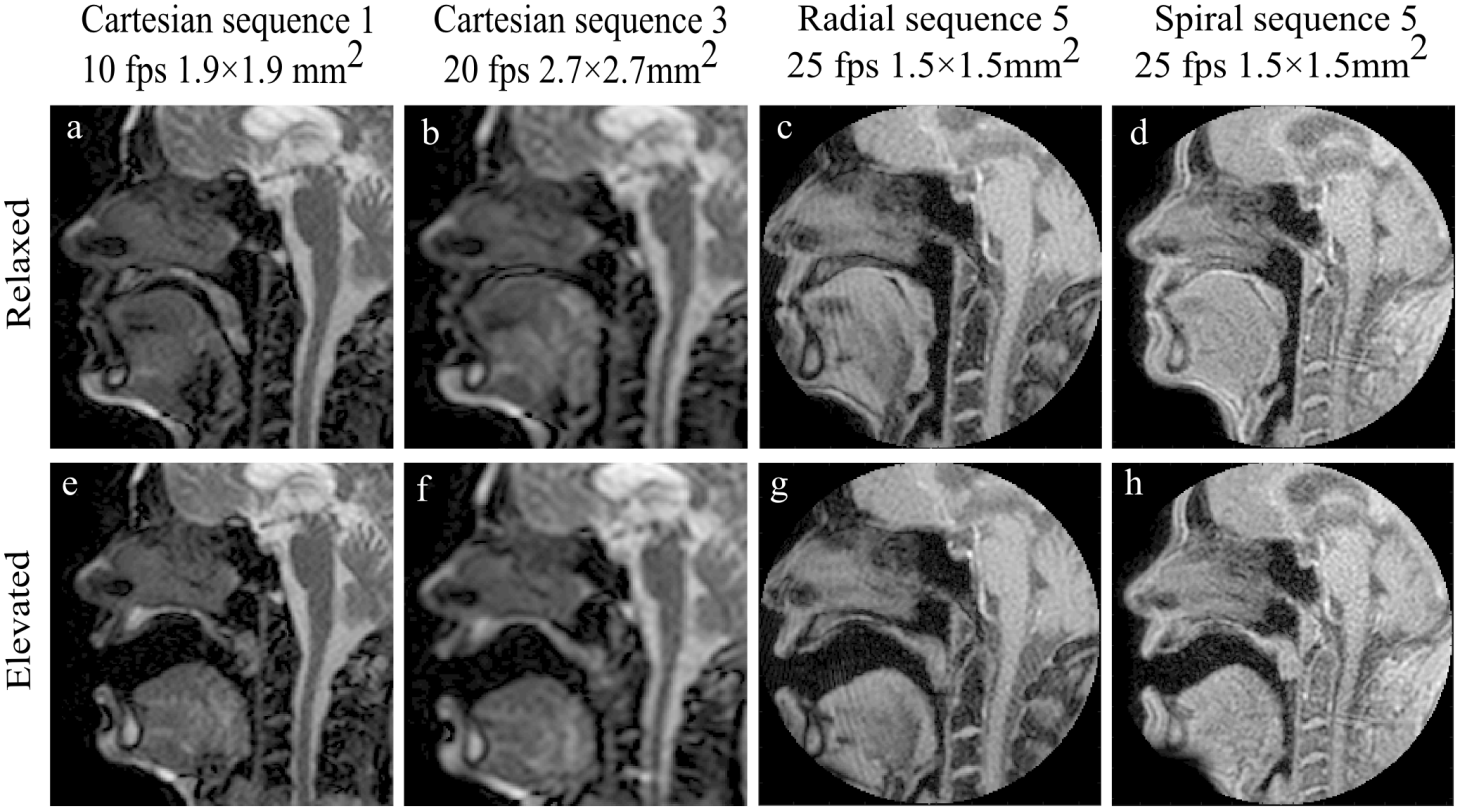
Example mid-sagittal images at elevated and relaxed velum positions acquired with sequence 1 and 3 Cartesian sampling and sequence 5 with radial and spiral acquisitions.

Example images acquired at both the elevated and relaxed velar positions are shown in Fig 4.

Measurements of velum thickness in millimeters at both velar positions are summarized in Table 2.

**Table 2:** Mean velum thickness and standard deviation in millimeters (mm) of all subjects measured in the relaxed (nasal breathing) and elevated (sustained phonation of /a/) velar positions.

Sequence	FLASH Radial		FLASH Spiral		bSSFP Cartesian		p-value
	Relaxed	Elevated	Relaxed	Elevated	Relaxed	Elevated	
1	8.7 (2.2)	11.5 (2.0)	8.7 (1.6)	11.7 (1.3)	9.6 (1.4)	12.3 (2.1)	0.65 ^NS^/0.77 ^NS^
2	9.1 (2.1)	12.0 (1.6)	8.7 (1.4)	12.3 (0.8)	9.9 (1.6)	12.1 (2.6)	0.59 ^NS^/0.95 ^NS^
3	9.5 (2.0)	12.2 (2.1)	9.3 (1.9)	12.6 (0.7)	10.0 (0.9)	12.2 (2.5)	0.75 ^NS^/0.92 ^NS^
4	9.0 (1.4)	11.3 (1.3)	8.5 (1.2)	11.4 (1.3)	-	-	0.56 ^NS^/0.86 ^NS^
5	9.3 (1.3)	10.5 (2.1)	8.6 (1.0)	10.3 (1.8)	-	-	0.39 ^NS^/0.83 ^NS^
p-value	0.95 ^NS^	0.66 ^NS^	0.93 ^NS^	0.06 ^NS^	0.86 ^NS^	0.99 ^NS^	

P-values refer to ANOVA analysis of velum thickness between sequences of the same sampling scheme (bottom row) and between sampling schemes within the same spatial-temporal compromise (right column, upper value corresponds to relaxed position and bottom value to elevated position). NS-not significant.

Mean velum thickness was determined as 9.15 ± 1.51 mm at the relaxed position and 11.73 ± 1.77 mm at the elevated position (p<0.0005). No significant difference (Table 2 bottom row) in velum thickness was found between sequences 1 to 5 for all sampling schemes at both the relaxed and elevated positions. Additionally, no significant difference (Table 2 right column) in velum thickness was found between sampling schemes (radial vs. spiral vs. Cartesian) at both velar positions.

Signal homogeneity of the velum measured at both velum positions is summarized in Table 3.

**Table 3:** Mean and standard deviation velum signal homogeneity measured from selected frames of the dynamic data at both the relaxed (nasal breathing) and elevated (sustained phonation of /a/) velar positions for all sequences.

Sequence	FLASH Radial		FLASH Spiral		bSSFP Cartesian		p-value
	Relaxed	Elevated	Relaxed	Elevated	Relaxed	Elevated	
1	4.03(0.40)	2.94(0.27)	4.93(0.95)	4.17(0.84)^c^	3.71(0.51)^a^	2.46(0.64)^a^	<0.005/<0.0005
2	4.50(0.77)	3.27(0.27)	4.63(1.07)	4.29(0.71)^c^	3.71(0.77)^d^	2.20(0.42)^a^ ^c^	<0.05/<0.0005
3	4.54(0.41)	3.76(0.55)	4.59(0.77)	3.55(0.77)	3.36(0.43)^a^ ^c^	2.29(0.41)^a^ ^b^	<0.0005 <0.0005
4	3.66(0.62)	2.84(0.38)	4.17(0.40)	3.66(0.51)	-	-	0.07 ^NS^/<0.01
5	3.59(0.50)	2.98(0.47)	3.75(0.52)	3.44(0.41)	-	-	0.59 ^NS^/0.06 ^NS^
p-value	<0.05	<0.05	<0.01	<0.05	0.14 ^NS^	0.30 ^NS^	

P-values refer to ANOVA analysis of signal homogeneity between sequences of the same sampling scheme (bottom row) and between sampling schemes within the same spatial-temporal compromise (right column, upper value corresponds to relaxed position and bottom value to elevated position). Post hoc Bonferroni paired t-test was used to identify statically significant pairs, where

^a^ p<0.0005 pairwise comparison to spiral acquisition.

^b^ p<0.0005 comparison to radial acquisition.

^c^ p<0.005 comparison to radial acquisition.

^d^ p<0.05 comparison to spiral.

NS—not significant.

It was observed that signal homogeneity of the velum was greater in the relaxed position for all sequences with Cartesian sampling (p<0.05). No significant difference in signal homogeneity was found between the relaxed and elevated positions for all sequences with spiral sampling. ANOVA analysis underlined significant differences between sequences for both velar positions for radial (p<0.05, Table 3 bottom row) and spiral (p<0.01 and p<0.05, Table 3 bottom row) acquisitions. Signal homogeneity in the relaxed position was found to be higher for sequence 1 when compared to sequence 5 (p<0.01) for the spiral acquisition. In the elevated position, there were significant differences in signal homogeneity between sequences 1 and 3 (p<0.05) and sequences 3 and 4 (p<0.05) for radial acquisition, and between sequences 2 and 5 (p<0.05) for spiral acquisition. However, no significant difference in signal homogeneity was found between sequences acquired with Cartesian sampling (Table 3 bottom row).

Significant pairs were found between spiral and Cartesian acquisitions for all sequences at both velar positions, and between radial and Cartesian for sequence 3 (relaxed position) and sequences 2 and 3 (elevated position). Comparison of non-Cartesian (spiral vs. radial) acquisitions revealed significant differences in velum signal homogeneity for sequences 1, 2 and 4 in the elevated position.

### Intensity-time CNR

**Fig 5:**
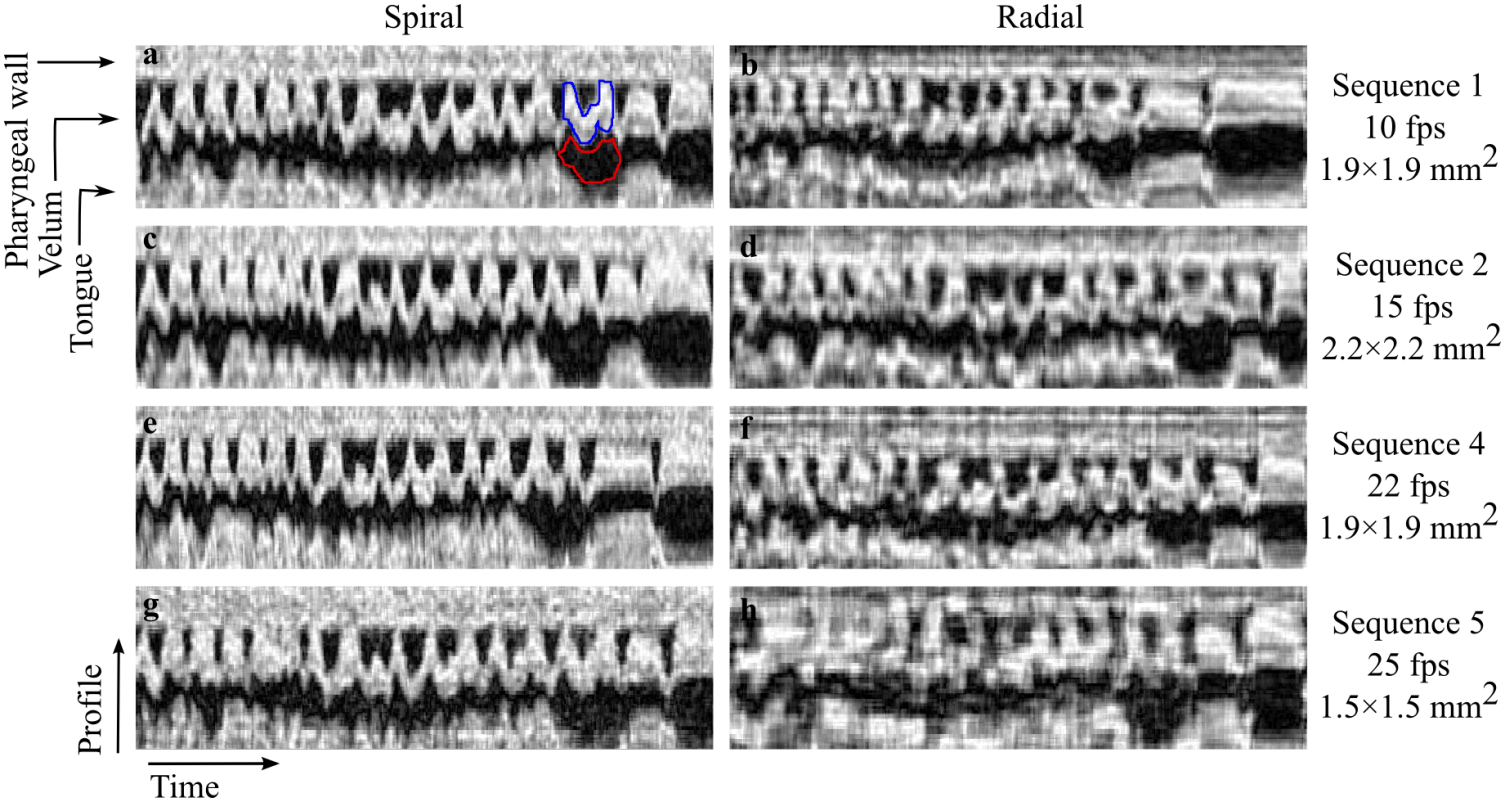
Intensity-time plots for spiral (a,c,e,g) and radial (b,d,f,h) acquisitions at different spatial-temporal resolution sets. Selected ROIs in the velum (blue) and in the neighboring oral cavity (red) were used to perform CNR measurements. At the highest frame rate of 25 fps (sequence 5), spiral acquisition shows adequate temporal fidelity (g) while radial acquisition shows temporal blurring and averaging of consecutive closure events (h).

Examples of intensity-time plots for radial and spiral acquisitions are shown in Fig 5. Increased temporal fidelity is noticeable in data acquired with spiral protocols (Fig 5a, 5c, 5e and 5g) when compared to the otherwise similar radial protocols (Fig 5b, 5d, 5f and 5h), particularly at higher frame rates. For example at 25 fps, data acquired with spiral sampling (Fig 5g) presents good distinction of closure events, as all points of contact between the velum and the pharyngeal wall are easily identified. However, intensity-time profiles acquired with the radial protocol showed increased temporal blurring and averaging of consecutive closure events (Fig 5h).

Intensity-time CNR measurements are summarized in Table 4.

**Table 4:** Mean and standard deviation CNR measured in a short section of the intensity-time plots.

Sequence	FLASH Radial	FLASH Spiral	bSSFP Cartesian	p-value
1	10.21 (1.74)	12.46 (1.31)	7.10 (1.87)^a^ ^c^	<0.005
2	12.51 (1.92)	13.81 (1.23)	6.67 (2.70)^b^ ^d^	<0.0005
3	13.27 (1.90)	17.68 (1.51)^a^	6.54 (2.71)^b^ ^d^	<0.0005
4	7.37 (1.02)	11.12 (0.59)	-	<0.0005
5	6.98 (1.09)	9.89 (0.94)	-	<0.005
p-value	<0.0005	<0.0005	0.93 ^NS^	

P-values refer to ANOVA analysis of CNR between sequences of the same sampling scheme (bottom row) and between sampling schemes within the same spatial-temporal compromise (right column). Post hoc Bonferroni paired t-test was used to identify statically significant pairs, where

^a^ p<0.05 pairwise comparison to radial.

^b^ p<0.005 pairwise comparison to radial.

^c^ p<0.005 pairwise comparison to spiral.

^d^ p<0.0005 pairwise comparison to spiral.

NS—not significant.

Comparison between sequences showed a CNR increase between sequence 1 and 3 for radial sampling (10.21±1.74 vs. 13.27 ± 1.90), however with borderline significance (p = 0.05) and for spiral sampling (12.46±1.31 vs. 17.68±1.51, p<0.0005). However, no significant change in CNR was found between sequences 1 to 3 (7.10±1.87 vs. 6.54±2.71, p = 0.93) for Cartesian sampling.

A decrease in CNR was observed for sequences 4 and 5, however with no significant difference (p = 1.00) between the two sequences for both radial and spiral acquisitions. In addition, comparison of sequence 4 with sequence 1 for both sampling methods (radial: 10.21±1.74 vs. 7.37±1.02, p = 1.02 and spiral: 12.46±1.31 vs. 11.12±0.59, p = 0.83) revealed no significant differences in CNR.

For sequences 1 to 3, non-Cartesian acquisitions provided higher CNR than the otherwise similar Cartesian acquisitions. At higher frame rates (sequences 4 and 5), spiral acquisitions provided higher CNR than equivalent radial protocols (refer to Table 4).

### Visual scoring

Intra-observer agreement was good (Cohen’s *k* = 0.59, p<0.0005) with differences in 20 out of 65 analyzed cases and a maximum intra-observer difference of 1 score point. Inter-observer agreement was very good (Cohen’s *k* = 0.74, p<0.0005) with differences between the observers in 13 of the 65 cases and a maximum inter-observer difference of 1 score point. In 11 of these 13 cases, observer 2 scored images higher by 1 point than observer 1. In 12 cases out of the 65, correspondent to data acquired with sequence 1 and 5 and spiral sampling, there was complete scoring agreement between the observers.

**Fig 6:**
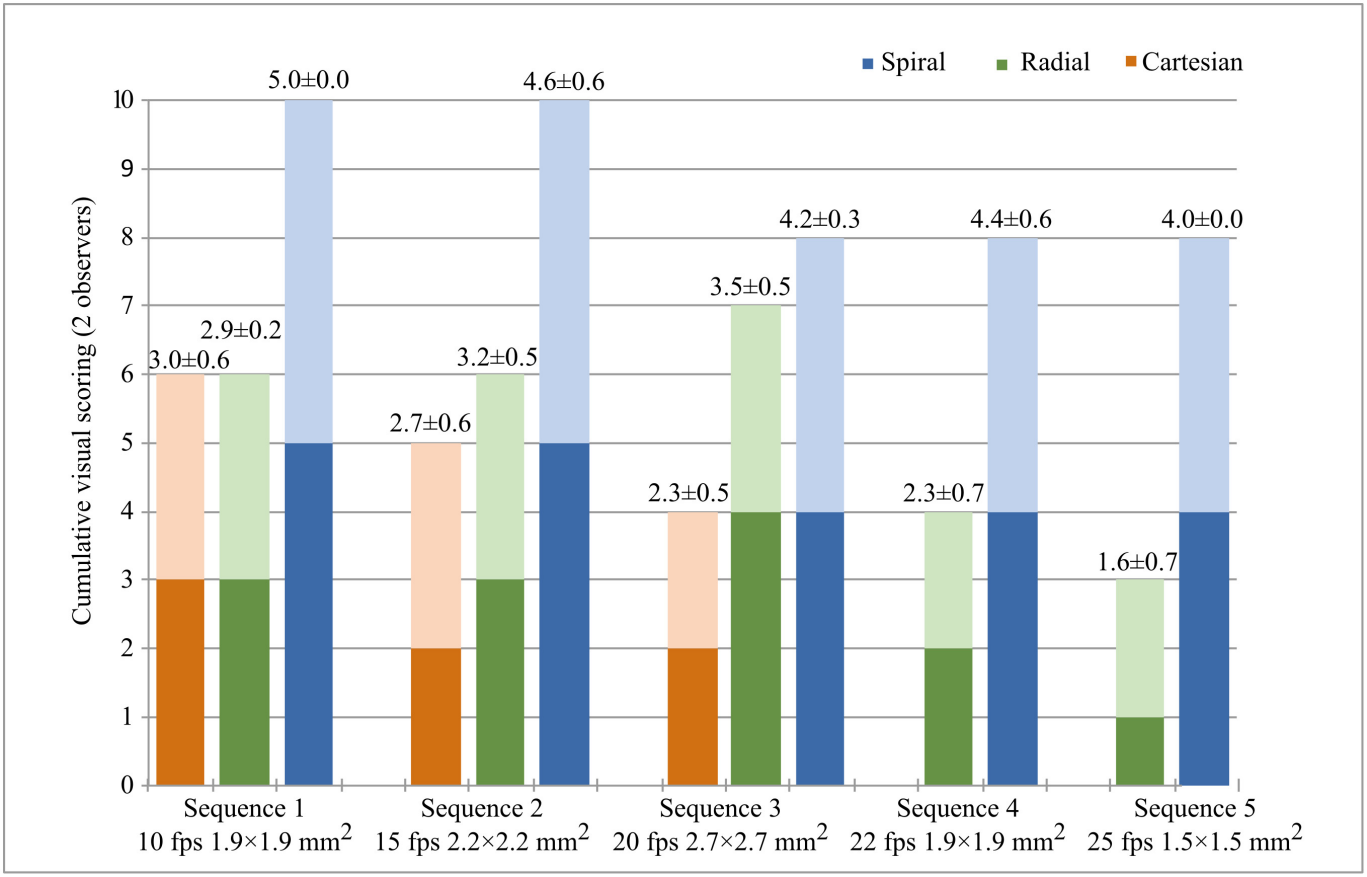
Histogram representation of the image quality cumulative visual scoring performed by 2 independent observers. Cumulative scoring represented by the sum of each observer independently (maximum scoring of 10) for all sequences and sampling schemes. Mean and standard deviation of visual scoring of both observers is presented numerically on top of each bar plot.

Histogram representation of visual scoring performed by the two observers is present in Fig 6.

Overall, spiral acquisitions provided superior image quality across sequences 1 to 3 than Cartesian acquisitions (p<0.01). Although visual scoring of data acquired with spiral sequence 1 presented superior image quality than the equivalent radial acquisition (5.0±0.0 vs. 2.9±0.2, p<0.01), no significant differences were found between the two sampling schemes for sequences 2 and 3. At higher frame rates, visual scoring of spiral data was superior to that of radial acquisitions for both sequence 4 (4.4±0.6 vs. 2.3±0.7, p<0.01) and sequence 5 (4.0±0.0 vs. 1.6±0.7, p<0.01). In total, 12% of the analyzed cases were scored as ‘Excellent’, all acquired with spiral sampling.

## Discussion

In the present study, we compared Cartesian and non-Cartesian real-time sequences at 1.5T, regarding image quality and temporal resolution trade-off, for dynamic speech imaging.

Previous studies [4,6,12,16] have performed dynamic imaging of velopharyngeal closure with clinically available hardware and Cartesian sequences in both healthy and VPI subjects. However, achieving sufficient temporal resolution to reliably assess VPI, while maintaining satisfactory image quality, is a difficult balance point to achieve and many studies have been limited to low frame rates. Although higher frame rates, 20 fps and upwards, with adequate image quality have been obtained with non-Cartesian acquisitions [9,11,25,28], these protocols are mostly reliant on off-line reconstruction methodologies and/or non-standard equipment, hampering immediate translation to clinical evaluation.

In this study, a non-Cartesian protocol optimized for fast dynamic imaging of the velum and velopharyngeal closure has been proposed. We were able to demonstrate differences in image quality and temporal resolution trade-off between FLASH non-Cartesian (spiral and radial) and bSSFP Cartesian imaging at 1.5T. The optimized non-Cartesian spiral protocol provided improved spatial-temporal resolution (22fps, 1.9×1.9×10 mm^3^ and 25 fps, 1.5×1.5×10 mm^3^) in comparison to Cartesian protocols, while still being easily implemented and reproducible with standard resources provided by the MRI scanner manufacturer.

Measured velum thickness was higher in the elevated velum position across all sequences. In agreement with previous literature [6,34], this was an expected result as the posterior-superior elevation of the velum leads to an increase in thickness in the sagittal plane.

Both Cartesian and radial acquisitions showed lower signal homogeneity of the velum in the elevated position across all sequences. As this measurement refers to the homogeneity of the signal intensity within the selected ROI, artifacts present in the velopharyngeal region, which distort and/or mask the velum, strongly reduce the measured parameter. The presence of artifacts in the velopharyngeal region in the elevated position can be seen in Fig 4e. Although both Cartesian and radial data present strong distortion of the velum in the elevated position, and consequently a decrease in the measured homogeneity, artifacts appear to be of different nature. Artifacts present in bSSFP Cartesian data appear to be due to off-resonance effects. This could be explained by that fact that when at rest, all velopharyngeal structures sit in close contact; however, during phonation, the elevation of the velum and separation of the velopharyngeal structures creates a larger area of tissue-air interface and bSFFP sequences are particularly sensitive to susceptibility differences. On the other hand, artifacts in radial data present a spokes-like pattern and are most likely due to radial under-sampling. However, by implementing a FLASH sequence with a spiral sampling, we were able to reduce artifacts or distortion in the region and improve overall signal homogeneity of the velum (Table 3) compared to the other two sampling methods.

Intensity-time CNR performance with non-Cartesian acquisitions was found to be superior to Cartesian acquisitions (Table 4). At higher frame rates, spiral protocols were optimal and provided higher CNR than equivalent radial protocols. Although no significant differences were found between Cartesian sequences 1 to 3, a gradual increase in CNR was found for radial and spiral acquisitions. As expected with the decrease in pixel size, a decrease in CNR follows for sequences 4 and 5 for both non-Cartesian. However, comparison between sequences 4 and 5 for both spiral and radial acquisitions revealed no significant difference, therefore indicating that improvements in spatial-temporal resolution (from 22 fps, 1.9×1.9×10 mm^3^ to 25 fps, 1.5×1.5×10 mm^3^) can be achieved with no significant loss in CNR. In addition, no significant difference in CNR was found between sequences 1 and 4 for both non-Cartesian acquisitions. This suggests that both non-Cartesian acquisitions allow doubling the temporal resolution, from 10 fps to 22 fps, while maintaining spatial resolution (1.9×1.9 mm^2^) and CNR. Although SSFP sequences commonly allow for superior image SNR than spoiled sequences like FLASH [5], this particular measurement of CNR reflects both the intrinsic signal-to-noise ratio and the presence of artifacts in the selected ROIs, i.e. the oral cavity. Thus, although spiral protocols were implemented with a FLASH-like acquisition, due to the reduced presence of artifacts in the velopharyngeal region, the measured CNR was in fact higher than that of bSSFP Cartesian acquisitions. This seems to suggest that the use of the FLASH spiral protocol should be preferred over the bSSFP Cartesian in this particular application, as an improvement in CNR and accurate distinction of the velum boundaries is possible without compromising spatial-temporal resolution.

Qualitative visual scoring provided additional insight on the overall image quality achieved by the different protocols. Overall, FLASH spiral data was scored higher than bSSFP Cartesian across all sequences. Although no significant difference was found between radial and spiral acquisitions at lower temporal resolutions, at higher frame rates of 22 and 25 fps, spiral acquisition was optimal in providing good visual image quality. Radial images, on the other hand, showed increased blurring and spokes-like artifacts, reducing the overall image quality scoring.

Differences between the chosen reconstruction algorithms must also be considered; while SENSE provides a true temporal acceleration of the Cartesian data, the “sliding window” method (non-Cartesian acquisitions) produces an interpolation of the data in time. In this case, since no additional information is being added to the raw data, temporal reliability is still dependent on the acquired frame rate and temporal smoothing is introduced. However, the study intended to compare different “best practice” protocols, i.e. that could be easily reproduced with resources routinely available and still provide adequate imaging. Since spiral acquisitions are intrinsically fast [35] and presented a superior native frame rate (about 6 fps) than radial, lower sliding window acceleration factors were required to achieve the desired frame rates than the equivalent radial acquisitions (Table 1). Radial acquisitions were less optimal with the current clinical setup, as these presented below optimal native frame rate (less than 2 fps) and therefore, blurring of the velum due to motion and averaging of consecutive closure events was present.

Limitations of the present work include the small sample size, as well as increased acoustic noise when using spiral protocols that may require additional care with more sensitive and/or younger subjects. However, noise cancellation and quality of audio was still optimal (supplementary data). Although peripheral nerve stimulation (PNS) clinical warning was displayed for some acquisitions, none of the scanned subjects reported PNS sensation.

## Conclusion

In conclusion, our results suggest that non-Cartesian real-time sequences are a promising tool to improve overall image quality and temporal resolution of dynamic speech imaging. We found that for all proposed sequences, FLASH non-Cartesian (spiral and radial) acquisitions provided higher CNR than bSSFP Cartesian acquisitions, within the same spatial-temporal resolution. With this clinical setup, FLASH spiral sequences were optimal and provided dynamic imaging with superior CNR, velum signal homogeneity and visual image quality. At temporal resolutions of 22 and 25 fps, spirals showed good temporal reliability of data while radial acquisitions showed increased temporal blurring and were less adequate. It should be underlined that it is possible to further improve image quality and temporal resolution of non-Cartesian acquisitions using alternative reconstruction methods and/or custom equipment and software [9,11,25,28,29]. However, the purpose of this study was to compare and present an easily reproducible protocol to researchers equipped with standard MRI resources and translatable to clinical practice.

## Supporting Information

S1 File Example video of rt-MRI of the upper vocal tract during speech acquired with Cartesian 10 fps sequence 1. (MP4)

S2 File Example video of rt-MRI of the upper vocal tract during speech acquired with Spiral 10 fps sequence 1. (MP4)

S3 File Example video of rt-MRI of the upper vocal tract during speech acquired with Cartesian 20 fps sequence 3. (MP4)

S4 File Example video of rt-MRI of the upper vocal tract during speech acquired with Spiral 22 fps sequence 4. (MP4)

S5 File Example video of rt-MRI of the upper vocal tract during speech acquired with Radial 22 fps sequence 4. (MP4)

S6 File Example video of rt-MRI of the upper vocal tract during speech acquired with Spiral 25 fps sequence 5. (MP4)
